# Using Period Analysis to Timely Assess and Predict 5-Year Relative Survival for Liver Cancer Patients From Taizhou, Eastern China

**DOI:** 10.3389/fonc.2022.920094

**Published:** 2022-07-04

**Authors:** Youqing Wang, Luyao Zhang, Fang Han, Runhua Li, Yongran Cheng, Xiyi Jiang, Liangyou Wang, Jinfei Chen, Jianguang Ji, Yuhua Zhang, Tianhui Chen

**Affiliations:** ^1^ Department of Cancer Prevention/Zhejiang Cancer Institute, Cancer Hospital of the University of Chinese Academy of Sciences (Zhejiang Cancer Hospital); Institute of Basic Medicine and Cancer (IBMC), Chinese Academy of Sciences, Hangzhou, China; ^2^ Department of Cancer Epidemiology and Prevention, Henan Engineering Research Center of Cancer Prevention and Control, Henan International Joint Laboratory of Cancer Prevention, The Affiliated Cancer Hospital of Zhengzhou University, Henan Cancer Hospital, Zhengzhou, China; ^3^ Department of Hepatobiliary and Pancreatic Surgery, Cancer Hospital of the University of Chinese Academy of Sciences (Zhejiang Cancer Hospital); Institute of Basic Medicine and Cancer (IBMC), Chinese Academy of Sciences, Hangzhou, China; ^4^ School of Public Health, Hangzhou Medical College, Hangzhou, China; ^5^ Department of Non-Communicable Chronic Disease Control and Prevention, Taizhou Center for Disease Control and Prevention, Taizhou, China; ^6^ Department of Oncology, The First Affiliated Hospital of Wenzhou Medical University, Wenzhou, China; ^7^ Center for Primary Health Care Research, Department of Clinical Sciences, Lund University, Malmö, Sweden

**Keywords:** 5-year relative survival, liver cancer, period analysis, cancer registry, eastern China

## Abstract

**Introduction:**

While timely assessment of long-term survival for patients with liver cancer is essential for the evaluation of early detection and screening programs of liver cancer, those data are extremely scarce in China. We aimed to timely and accurately assess long-term survival for liver cancer patients in eastern China.

**Methods:**

Patients diagnosed with liver cancer during 2004–2018 from four cancer registries with high-quality data from Taizhou, eastern China, were included. The period analysis was used to calculate the 5-year relative survival (RS) for overall and the stratification by sex, age at diagnosis, and region. The projected 5-year RS of liver cancer patients during 2019–2023 was also assessed using a model-based period analysis.

**Results:**

The overall 5-year RS for patients with liver cancer during 2014–2018 reached 32.4%, being 29.3% for men and 36.1% for women. The 5-year RS declined along with aging, decreasing from 38.2% for age <45 years to 18.8% for age >74 years, while the 5-year RS for urban area was higher compared to rural area (36.8% vs. 29.3%). The projected overall 5-year RS of liver cancer patients could reach 41.4% during the upcoming period 2019–2023.

**Conclusions:**

We provided, for first time in China using the period analysis, the most up-to-date 5-year RS for patients with liver cancer from Taizhou, eastern China, and also found that the 5-year RS for liver cancer patients have improved greatly during 2004–2018, which has important implications for the timely evaluation of early detection and screening programs for patients with liver cancer in eastern China.

## Introduction

Liver cancer ranks as the sixth most common cancer and the third leading cause of cancer death globally according to the latest data of GLOBOCAN 2020 (1). While, overall, 108,081 new cases and 94,213 deaths of liver cancer occurred in 2016 across China from the National Cancer Registry Annual Report 2019 (2), liver cancer ranked as the fourth most common cancer and the second leading cause of cancer death during 2010–2016 in Taizhou, eastern China, with the crude incidence rate reaching 35.16/10^5^ and the crude mortality rate reaching 33.78/10^5^ (3).

Long-term survival estimates assessed using population-based cancer registry data are essential for the evaluation of cancer burden. The 5-year relative survival (RS) is the most important index assessing cancer burden and is essential for the evaluation of early detection and screening programs for the majority of cancer types, which shall be as up-to-date as possible. The assessment of the 5-year RS has been commonly used by cohort, complete, and period approaches (4). Nevertheless, because traditional cohort and complete methods have to use 5-year follow-up data, the approaches will delay 5 years at least for survival estimates (in addition to other time requests for data collection, calculation, and publication). The period analysis, which does not require 5-year follow-up data to calculate survival estimates, is the “gold standard” for the assessment of the long-term survival of cancer patients using data from population-based cancer registries and has been widely used in Western populations (5, 6). Later in 2006, Brenner and Hakulinen (7) proposed a model-based period analysis approach using the generalized linear model, which could forecast future survival during an upcoming period (8). However, the application of the period approach in China has been scarce.

Our group found, for the first time, by systematically using the period analysis and cancer registry data from eastern China, that the period analysis is superior to traditional cohort and complete methods, which can provide more up-to-date precise estimates of the long-term survival for overall and the stratification by sex, age at diagnosis, region, and cancer sites (9). For instance, for liver cancer, during 2009–2013, the 5-year RS derived from the period analysis was found to be much closer to the observed actual survival compared to those derived from complete and cohort methods. Thus, the period analysis performed better than the traditional method for liver cancer patients.

In this study, we aimed to provide the most up-to-date (during 2014–2018) estimates of the 5-year RS for liver cancer patients from the Chinese population using the period analysis and population-based cancer registry data from Taizhou city, eastern China. We also aimed to assess the trends of the 5-year RS during 2004–2018 and to forecast the 5-year RS for the upcoming 2019–2023 period using the data during 2004–2018 and a model-based period analysis.

## Materials and Methods

### Data Sources

Taizhou city with 6.6 million inhabitants is located at the eastern coast of Zhejiang province and approximately 300 km away from southern Shanghai, China. The Information and Management System for Zhejiang Provincial Chronic Disease Surveillance was established in 2001 as a platform for monitoring the incidence and mortality rates of chronic diseases (including cancer) for inhabitants living in Zhejiang province (10). This population-based cancer surveillance system was used to assess the incidence rates for cancer from nine registries in Taizhou. The proportion of death certificate only (DCO) cases as a fraction of total cases was used to judge the quality of these data, with <13% being considered acceptable (7). In light of these criteria, data from four (Luqiao, Yuhuan, Xianju, and Wenling) out of nine registries from Taizhou region were included for further analyses.

The International Classification of Diseases, 10th Revision (ICD-10), and the third edition of the International Classification of Diseases for Oncology (ICD-O-3) were used for cancer coding. Liver cancer patients (using the ICD-10 code C22) diagnosed between January 1, 2004, and December 31, 2018, were included, while follow-up information was collected through December 31, 2018, using a combination of passive and active methods. Overall, 11,519 liver cancer patients were initially identified, and among them, 664 were lost to follow-up, 136 were unknown cases, and 2,361 were missing at the last follow-up, which were eventually excluded. Thus, 8,250 patients during 2004–2018 were included for further analyses.

### Statistical Analysis

The distribution differences in the basic characteristics of the patients were compared among the three periods of 2004–2008, 2009–2013, and 2014–2018. Data were analyzed using the χ^2^ test for categorical variables. A *P* value <0.05 was considered to be statistically significant.

Throughout this article, the 5-year RS estimates for liver cancer patients were calculated as the ratio of the observed survival in the patient group with liver cancer and the expected survival from a comparable group in the general population (11). The expected survival was derived from life tables for the four cities (Luqiao, Wenling, Xianju, and Yuhuan) of the Taizhou population stratified by sex, age, region, and calendar year using the Ederer II approach.

The period approach was used to calculate the 5-year RS for patients during the 2014–2018 interval. All of the patients were separated into two subsets, i.e., those who were newly diagnosed from 2014 to 2018 and those who had been diagnosed prior to this period (2009–2013) who remained alive within this period. The period analysis was used to deal with left-censored data diagnosed prior to the period of interest and right-censored data corresponding to patients remaining alive at the end of the interest period. Using this approach, data were compiled to generate a life table, with 1-year RS (*S_i_*) at year *i* of follow-up being calculated as follows:


$$S_{i}=1-\frac{{\text{d}}_{i}}{n_{i}-c_{i}/2}$$

Where *n*_i_ corresponds to the population at the start of year *i* of follow-up, *d_i_* corresponds to the number of deaths at the end of year *i*, and *c_i_* corresponds to the number of censored data in year *i*.

The observed survival (*S_k_*) values for k-years were determined by multiplying by the k-year conditional 1-year survival rate as follows:


$$\bar{S_{k}}=\prod_{i=1}^{k}S_{i}$$

RS was defined as the ratio of observed to expected survival and was calculated as follows:


$$R_{i}=\frac{\bar{S_{k}}}{S_{k}^{*}}$$

Where *k* was 5 when calculating 5-year RS, $\bar{S_{k}}$ corresponded to observed survival, and $S_{k}^{*}$ corresponded to expected survival. RS estimates and corresponding standard error (*SE*) values were calculated as per the Greenwood method (6).

Model-based period analyses were then used to predict the 5-year RS rates for liver cancer patients over the 2019–2023 period, with additional patient stratification based on age at diagnosis, sex, and region. For these analyses, data from the 2004–2008, 2009–2013, and 2014–2018 intervals were included, with follow-up year and conditional 1-year survival rates for each year, respectively, serving as independent and dependent variables. The 5-year RS over the 2019–2023 period was then predicted by establishing a generalized linear model (GLM) *via* binomial regression.

The “periodR” package for R version 3.13 (R Foundation for Statistical Computing, Vienna, Austria) was used for all statistical analyses (7).

## Results

### Basic Characteristics of Liver Cancer Patients

The basic characteristics of liver cancer patients are presented in **Table 1**. Overall, 8,250 liver cancer patients were included, and the number of patients increased over 2004–2018. While more men were found compared to women (6,422 men and 1,828 women), the male-to-female sex ratio reached 3.5 for overall and over 2004–2018, but no difference in sex distribution was found over 2004–2018 (*P* = 0.94). Rural areas had more patients compared to urban areas (89% vs. 11%), and this difference reached statistical significance over 2004–2018 (*P* < 0.001). Overall, the average age at diagnosis reached 61.5 years, age at diagnosis <45 years accounted for only 9%, and >50% of the patients ranged 55–74 years, while over the 2004–2008 period, this distribution was retained and reached statistical significance (*P* < 0.001).

**Table 1:** Basic characteristics of liver cancer patients diagnosed during 2004–2018 in Taizhou, eastern China.

Characteristics	Number of cases (%)	Diagnosed interval			*P*
		2004–2008	2009–2013	2014–2018	
**Total**	8,250 (100)	1,516 (100)	2,941 (100)	3,793 (100)	
**Sex**					
** Male**	6,422 (77.8)	1,181 (77.9)	2,283 (77.6)	2,958 (78.0)	0.9381
** Female**	1,828 (22.2)	335 (22.1)	658 (22.4)	835 (22.0)
**Male-to-female ratio**	3.51	3.53	3.47	3.54	
**Region**					
**Urban area**	904 (11.0)	12 (0.8)	366 (12.4)	526 (13.9)	<0.001
**Rural area**	7,346 (89.0)	1,504 (99.2)	2,575 (87.6)	3,267 (86.1)
**Average age (years)**	61.5	60.7	61.2	61.7	
**Age at diagnosis (years)**					
**<45**	748 (9.1)	202 (13.3)	278 (9.5)	268 (7.1)	<0.001
**45–54**	1,831 (22.2)	317 (20.9)	667 (22.7)	847 (22.3)
**55–64**	2,389 (28.9)	382 (25.2)	882 (30.0)	1,125 (29.7)
**65–74**	1,944 (23.6)	378 (24.9)	680 (23.1)	886 (23.3)
**>74**	1,338 (16.2)	237 (15.6)	434 (14.7)	667 (17.6)

### Five-Year Relative Survival of Liver Cancer Patients During 2014–2018

As shown in **Table 2**, we found that the 5-year RS during 2014–2018 reached 32.4% and women had a higher 5-year RS compared to that in men (36.1% vs. 29.3%). We found a clear age gradient for the 5-year RS, declining from 38.2% for age at diagnosis <45 years to 18.8% for age >74 years. Urban areas had a higher 5-year RS compared to that in rural areas (36.8% vs. 29.3%).

**Table 2:** Survival of liver cancer patients during 2014–2018 in Taizhou, eastern China.

	Estimated value (%)	Standard error (SE)
**Total**	32.4	0.7
**Sex**		
**Men**	29.3	0.9
**Women**	36.1	1.3
**Age at diagnosis (years)**		
**<45**	38.2	2.4
**45–54**	35.4	1.6
**55–64**	29.2	0.7
**65–74**	19.3	0.5
**>74**	18.8	1.5
**Region**		
**Urban area**	36.8	2.3
**Rural area**	29.3	0.7

### Prediction During the Upcoming 2019–2023 and Trends in the 5-Year Relative Survival During 2004–2023

**Figure 1:**
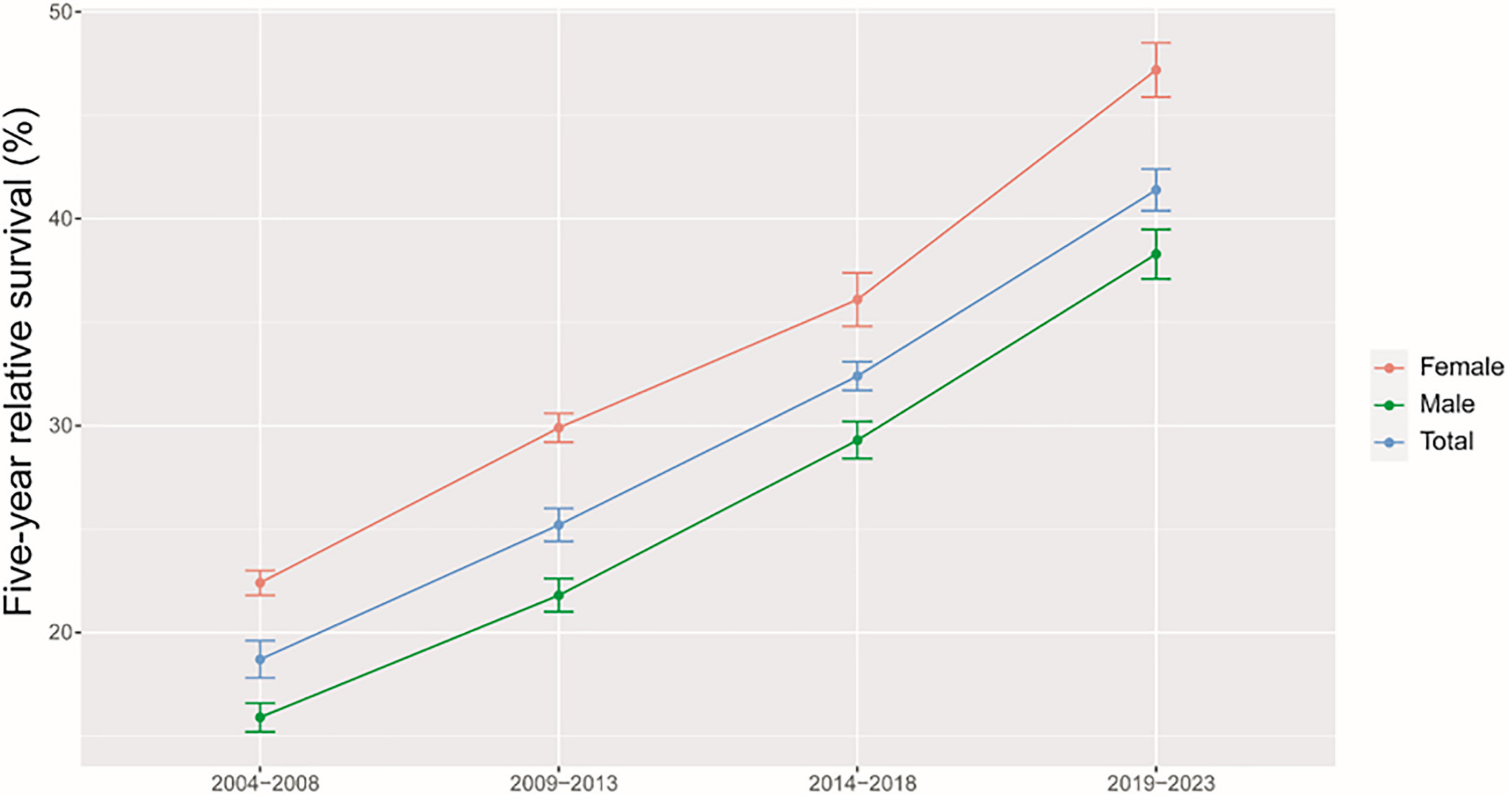
Five-year relative survival for total, male and female liver cancers in 2004–2008, 2009–2013, 2014–2018, and 2019–2023.

**Figure 2:**
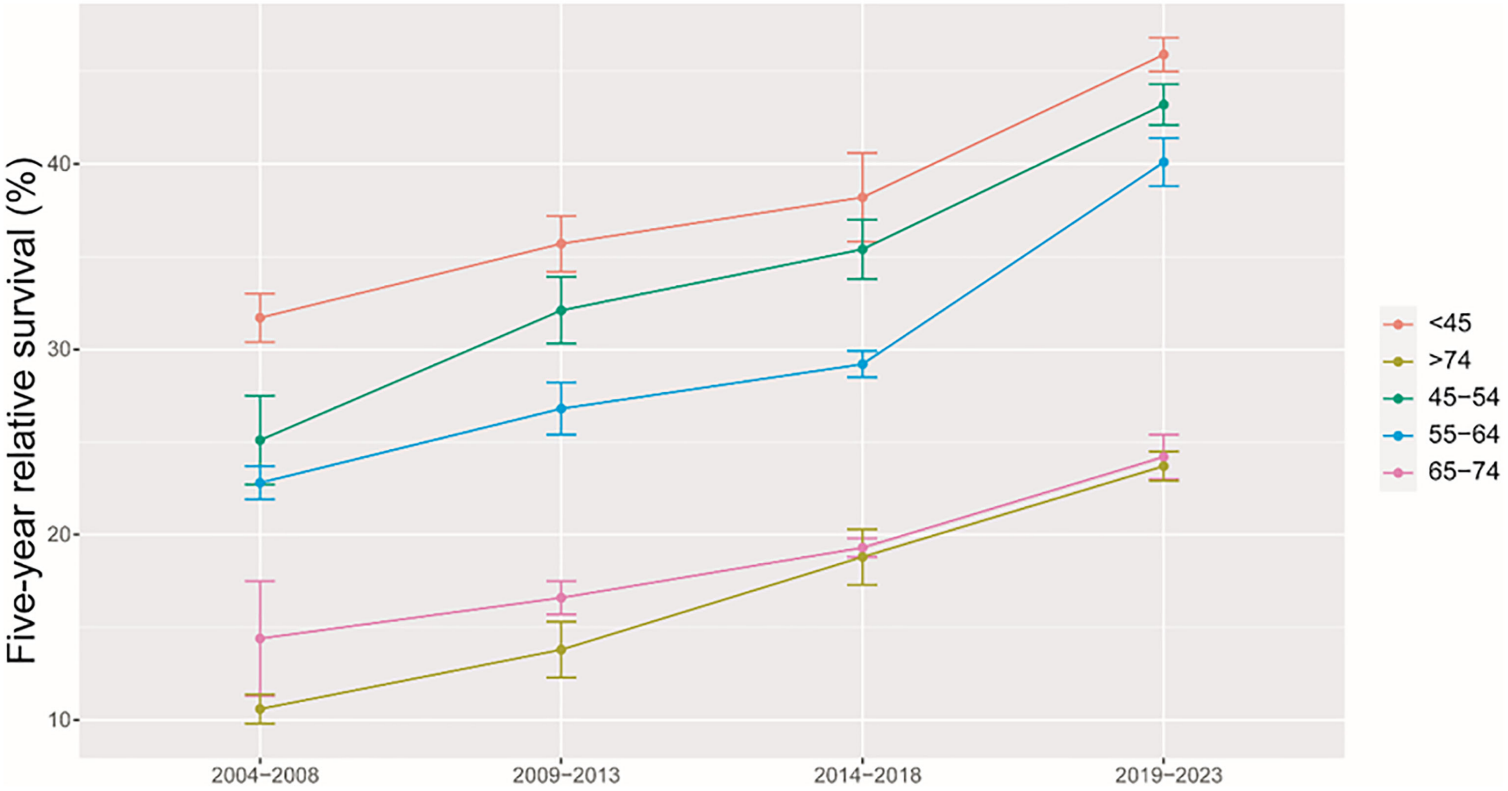
Five-year relative survival of liver cancers for different ages at diagnosis in 2004–2008, 2009–2013, 2014–2018, and 2019–2023.

**Figure 3:**
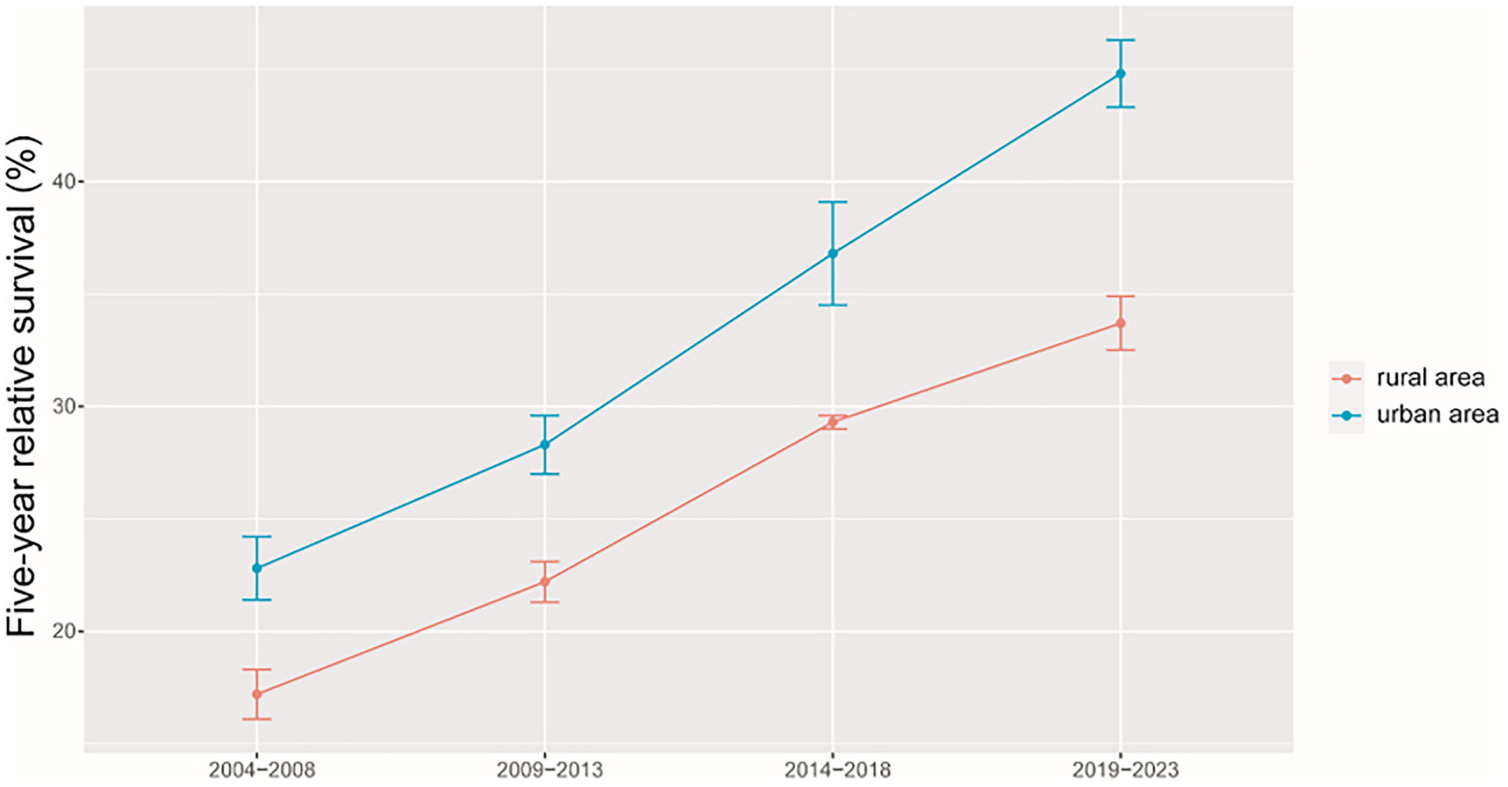
Five-year relative survival of liver cancers for urban and rural areas in 2004–2008, 2009–2013, 2014–2018, and 2019–2023.

As shown in **Table 3**, we predicted that the overall 5-year RS during the upcoming period could reach 41.4% (38.3% for men and 47.2% for women) using three continuous 5-year (2004–2008, 2009–2013, and 2014–2018) data on survival and a model-based period analysis. We also found a clear age gradient for the 5-year RS, declining from 45.9% for age at diagnosis <45 years to 23.7% for age >74 years. Urban areas had a higher 5-year RS compared to that in rural areas (44.8% vs. 37.7%). We found a clear increasing trend in the 5-year RS during 2004–2023 for overall and the stratification by sex (**Figure 1**), age at diagnosis (**Figure 2**), and region (**Figure 3**).

**Table 3:** Prediction of the survival of liver cancer patients during 2019–2023 in Taizhou, eastern China.

	Estimated value (%)
**Total**	41.4
**Sex**	
** Men**	38.3
** Women**	47.2
**Age at diagnosis (years)**	
** <45**	45.9
** 45–54**	43.2
** 55–64**	40.1
** 65–74**	24.2
** >74**	23.7
**Region**	
** Urban area**	44.8
** Rural area**	37.7

## Discussion

We provided, for the first time in China using the period analysis, the most up-to-date 5-year RS for patients with liver cancer from Taizhou, eastern China, reaching 32.4% for overall during 2014–2018. Women had a higher 5-year RS compared to that in men (36.1% vs. 29.3%), and urban areas had a higher 5-year RS compared to that in rural areas (36.8% vs. 29.3%). We also found a clear age gradient for the 5-year RS, declining from 38.2% for age at diagnosis <45 years to 18.8% for age >74 years. Additionally, we predicted that the overall 5-year RS during the upcoming period could reach 41.4% (38.3% for men and 47.2% for women) using three continuous 5-year (2004–2008, 2009–2013, and 2014–2018) data on survival and a model-based period analysis. We found a clear increasing trend in the 5-year RS during 2004–2023 for overall and the stratification by sex, age at diagnosis, and region.

Liver cancer ranks as the second leading cause of cancer death in Taizhou, eastern China (3). Our finding of the 5-year RS reaching 32.4% during 2014–2018 for Taizhou is higher than the report of 19.1% during 2005–2010 for Zhejiang province (10) and is also higher than the report of 12.1% during 2012–2015 for China (11). Nevertheless, our result is plausible due to the following reasons. First, the calculation period was 8 years earlier for the report of 19.1% during 2005–2010 for Zhejiang province compared to our data (2014–2018). It is common sense that the survival of liver cancer patients shall be improved along with the progress of science and technology and the improvement of treatment over recent years (8 years later for our data) (12–16), as well as the implementation of early screening programs for liver cancer patients (17–20) and the accessibility of medical insurance systems for the local population. Second, the report of 19.1% for 2005–2010 was calculated by the cohort approach, which shall be significantly lower compared to the survival estimate calculated by the period approach, as confirmed by our group for the 5-year RS during 2009–2013 (9). Third, the report of 12.1% during 2012–2015 for China was actually projected rather than estimated. Because the data for the study were from 17 cancer registries only with cancer patients diagnosed until the end of 2013 and followed up until the end of 2015 (11), the 5-year RS for patients with any cancer type including liver cancer could be calculated at the latest for 2013, while the survival data after 2013 could only be projected, which may be biased by existing data. Taken together, our data could be significantly higher, considering that our data source was from eastern China with qualified data and an advanced health care system compared to the data on the 17 cancer registries from varied health care systems (11).

Our finding of women with a higher 5-year RS compared to that in men (36.1% vs. 29.3%) is plausible because men have a higher possibility of alcohol consumption, tobacco smoking, and hepatitis B virus (HBV)/hepatitis C virus (HCV) infection (21–24) compared to that in women. Prior studies have confirmed liver cancer incidence and mortality rates to be higher among men relative to those in women, with men exhibiting worse survival, which could be attributed to the protective effects of estrogens (21, 25–27). We also found that the 5-year RS rate for urban areas was higher than that in rural areas (36.8% vs. 29.3%), which may be mainly attributed to differences between rural and urban populations with respect to socioeconomic status, medical/health resource allocation, and/or health education (28–30). Additionally, the limited numbers of medical professionals and outdated equipment in rural areas can lead to the further aggravation of resource allocation in rural areas. Government officials should thus pay further attention to equity as a function of geographic area, guiding patients toward rational treatment selection while optimizing resource investment in these different areas. We observed a clear age gradient for the 5-year RS, declining from 38.2% for age at diagnosis <45 years to 18.8% for age>74 years, indicating that survival rates are higher among young patients, in line with other reports (31, 32) and with the common sense that overall liver functionality shall be better in younger individuals compared to that in the elderly.

Our findings of a clear increasing trend in the 5-year RS during 2004–2023 for overall and the stratification by sex, age at diagnosis, and region are also plausible due to the following reasons. Firstly, while advances in clinical treatment approaches have been beneficial to liver cancer patient survival, surgical resection remains the most beneficial treatment associated with improvements in survival for the liver cancer patient (12–15). Second, B-ultrasound and alpha fetoprotein (AFP)-based screening approaches have been used in routine physical examinations over recent years, enabling the potential detection of abnormalities in individuals who are otherwise largely asymptomatic (18–20). Therefore, substantial improvements in survival for the liver cancer patient over the past 10 years could be likely attributable to improvements in surveillance, screening, and early detection. Third, Taizhou, being a coastal city in Zhejiang Province, eastern China, has a rapidly growing economy and a wide coverage of medical insurance system. The improvements in survival for liver cancer patients over time may be also attributable to the highly educated nature of the Taizhou population, since the awareness rates about core knowledge of cancer prevention in Zhejiang is reaching 75% (32), given that these individuals may be more aware of the importance of physical health as compared to individuals from other regions.

Our study has a number of strengths and limitations. Three strengths are listed below. First, we provided, for the first time in China using the period analysis, the most up-to-date (during 2014–2018) 5-year RS for patients with liver cancer from Taizhou, eastern China. Second, we assessed the survival trends and found that the 5-year RS for liver cancer patients has improved greatly during 2004–2008, 2009–2013, and 2014–2018. Third, we projected the upcoming 5-year RS during 2019–2023. We also have limitations. First, we could not provide stratified survival data on stage, histology, and treatment of liver cancer patients. Nevertheless, population-based cancer registries commonly do not include clinical information on stage (such as TNM), histology, and treatment of cancer patients. Hopefully, hospital-based cancer registries including detailed information on cancer patients will be available soon in the near future. Second, we only provided the most up-to-date survival data for liver cancer patients from Taizhou, eastern China. Therefore, further investigations using provincial or national data are also highly warranted.

## Conclusion

In this study, we provided, for the first time in China using the period analysis, the most up-to-date 5-year RS for patients with liver cancer. Overall, the results of this study show that the overall 5-year RS among liver cancer patients has gradually increased, regardless of gender, age at diagnosis, and region at diagnosis. Our timely data on 5-year RS for liver cancer patients from Taizhou, eastern China, are essential for the evaluation of early detection and screening programs for liver cancer in Taizhou, eastern China.

## Data Availability Statement

The raw data supporting the conclusions of this article will be made available upon reasonable request to the corresponding authors.

## Ethics Statement

Although the data from the nine cancer registries from Taizhou City, eastern China, were completely anonymous and their use did not entail ethical problems, ethical approval by the institutional review board of Zhejiang Cancer Hospital, China, was obtained.

## Author Contributions

TC was responsible for the study concept and design. TC obtained funding. LW and TC acquired the data. YC analyzed and interpreted the data. YW, LZ, FH, JC, JJ, YZ, and TC drafted the article, and all authors revised it for important intellectual content. All authors contributed to the article and approved the submitted version.

## Funding

This work was supported by grants from the National Key Research-Development Program of China (2021YFC2500400 and 2021YFC2500401), Zhejiang Provincial Ten-Thousand Talents Plan (2021R52020), and Joint Key Program of Zhejiang Province-Ministry of Health (WKJ-ZJ-1714). The funding agencies had no role in the design and conduct of the study; collection, management, analysis, and interpretation of the data; preparation, review, or approval of the article; and decision to submit the article for publication.

## Conflict of Interest

The authors declare that the research was conducted in the absence of any commercial or financial relationships that could be construed as a potential conflict of interest.

## Publisher’s Note

All claims expressed in this article are solely those of the authors and do not necessarily represent those of their affiliated organizations, or those of the publisher, the editors and the reviewers. Any product that may be evaluated in this article, or claim that may be made by its manufacturer, is not guaranteed or endorsed by the publisher.
